# Machine learning algorithms using national registry data to predict loss to follow-up during tuberculosis treatment

**DOI:** 10.1186/s12889-024-18815-0

**Published:** 2024-05-23

**Authors:** Moreno M. S. Rodrigues, Beatriz Barreto-Duarte, Caian L. Vinhaes, Mariana Araújo-Pereira, Eduardo R. Fukutani, Keityane Bone Bergamaschi, Afrânio Kristki, Marcelo Cordeiro-Santos, Valeria C. Rolla, Timothy R. Sterling, Artur T. L. Queiroz, Bruno B. Andrade

**Affiliations:** 1 Multinational Organization Network Sponsoring Translational and Epidemiological Research (MONSTER) Initiative, Salvador, Brazil; 2 Laboratório de Análise e Visualização de Dados, Fundação Oswaldo Cruz, Porto Velho, Brazil; 3 Programa de Pós-Graduação em Clínica Médica, Universidade Federal do Rio de Janeiro, Rio de Janeiro, Brazil; 4 Instituto de Pesquisa Clínica e Translacional, Curso de Medicina, Salvador,Faculdade ZARNS,, Brazil; 5 Laboratório de Pesquisa Clínica e Translacional, Instituto Gonçalo Moniz, Fundação Oswaldo Cruz, Salvador, Brazil; 6 Departamento de Infectologia, Hospital das Clínicas da Faculdade de Medicina da Universidade de Sao Paulo,, Sao Paulo, Brazil; 7 Curso de Medicina, Escola Bahiana de Medicina e Saúde Pública, Salvador, Brazil; 8 Faculdade de Medicina, Universidade Federal da Bahia, Salvador, Brazil; 9 Programa Acadêmico de Tuberculose da Faculdade de Medicina, Universidade Federal do Rio de Janeiro, Rio de Janeiro, Brazil; 10 Fundação Medicina Tropical Doutor Heitor Vieira Dourado, Manaus, Brazil; 11 Faculdade de Medicina, Universidade Nilton Lins, Manaus, Brazil; 12 Laboratório de Pesquisa Clínica em Micobacteriose, Instituto Nacional de Infectologia Evandro Chagas, Fiocruz, Rio de Janeiro, Brazil; 13 Division of Infectious Diseases, Department of Medicine, Vanderbilt University School of Medicine, Nashville, TN USA; 14 Laboratório de Análise de Visualização de Dados, FIOCRUZ Rondônia, Rua da Beira, Laoga, Porto Velho, Rondônia 7617, 76812-245 Brazil; 15 Laboratório de Inflamação e Biomarcadores, Instituto Gonçalo Moniz, Fundação Oswaldo Cruz, Rua Waldemar Falcão, 121, Candeal, Salvador, Bahia 40296-710 Brazil

**Keywords:** Tuberculosis, Score prediction, Loss to follow-up, Machine learning

## Abstract

**Background:**

Identifying patients at increased risk of loss to follow-up (LTFU) is key to developing strategies to optimize the clinical management of tuberculosis (TB). The use of national registry data in prediction models may be a useful tool to inform healthcare workers about risk of LTFU. Here we developed a score to predict the risk of LTFU during anti-TB treatment (ATT) in a nationwide cohort of cases using clinical data reported to the Brazilian Notifiable Disease Information System (SINAN).

**Methods:**

We performed a retrospective study of all TB cases reported to SINAN between 2015 and 2022; excluding children (< 18 years-old), vulnerable groups or drug-resistant TB. For the score, data before treatment initiation were used. We trained and internally validated three different prediction scoring systems, based on Logistic Regression, Random Forest, and Light Gradient Boosting. Before applying our models we splitted our data into training (~ 80% data) and test (~ 20%) sets, and then compared the model metrics using the test data set.

**Results:**

Of the 243,726 cases included, 41,373 experienced LTFU whereas 202,353 were successfully treated. The groups were different with regards to several clinical and sociodemographic characteristics. The directly observed treatment (DOT) was unbalanced between the groups with lower prevalence in those who were LTFU. Three models were developed to predict LTFU using 8 features (prior TB, drug use, age, sex, HIV infection and schooling level) with different score composition approaches. Those prediction scoring systems exhibited an area under the curve (AUC) ranging between 0.71 and 0.72. The Light Gradient Boosting technique resulted in the best prediction performance, weighting specificity and sensitivity. A user-friendly web calculator app was developed (https://tbprediction.herokuapp.com/) to facilitate implementation.

**Conclusions:**

Our nationwide risk score predicts the risk of LTFU during ATT in Brazilian adults prior to treatment commencement utilizing schooling level, sex, age, prior TB status, and substance use (drug, alcohol, and/or tobacco). This is a potential tool to assist in decision-making strategies to guide resource allocation, DOT indications, and improve TB treatment adherence.

**Supplementary Information:**

The online version contains supplementary material available at 10.1186/s12889-024-18815-0.

## Introduction

Despite the widespread availability of curative treatment of tuberculosis (TB), this disease remains a major plague of humanity, accounting for more than one million deaths annually [1]. Global treatment success is still below the targets established by the World Health Organization (WHO) [2, 3], especially in low- and middle-income countries (LMIC) such as Brazil [4].

Current WHO treatment recommendations for drug-susceptible TB include six months of a combination of antibiotics [3]. Such long treatment is associated with an increased risk of loss to follow up (LTFU) and may lead to adverse drug reactions [2]. Early identification of patients at high risk of LTFU at the moment of diagnosis with clinical and sociodemographic characteristics is key to providing personalized care, which may involve directly observed treatment (DOT), and helping decision-making strategies to mitigate losses in the cascade of care. Noteworthy, the Brazilian Ministry of Health recommends DOT for all TB cases, but the rates of cases that carry out the DOT still represent less than 50% of the total cases reported. To do so, the establishment of reliable and accurate prediction tools [4] is necessary, especially when limited resources require prioritization of intensive case management tools with a high-middle TB disease burden .

Brazil is among the countries with the highest number of TB cases in the world, despite the fact that it follows the WHO’s standardized TB treatment recommendations. Importantly, the cascade of care in Brazil for drug-sensitive TB is composed of 3 steps: (1) mandatory reporting of TB cases to the Notifiable Diseases Information System (SINAN) [5, 6]; (2) a six-month treatment regimen, usually in fixed-dose combination (FDC) [7]; and (3) treatment-associated outcomes are reported in the SINAN database. Thus, this is a significant source of data that could be explored to develop prediction models for LTFU during anti-TB treatment (ATT).

Therefore, we aimed to develop a web-based prediction model for LTFU among pulmonary TB treatment cases in Brazil at the baseline consultation utilizing secondary data elements readily available at diagnosis. Importantly, the developed a model that could be used by both the Brazilian government and clinicians as a readily available web-based tool for decision-making to achieve higher rates of TB treatment success.

## Materials and methods

### Ethics statement

All data accessed in this study were obtained from a publicly available platform and pre-processed by the Brazilian Ministry of Health (https://datasus.saude.gov.br) This processing verified the data regarding consistency, duplicate registration, and completeness, following the instructions set by Resolution Number 466/12 on Research Ethics of the National Health Council, Brazil. There was no identifiable information in the databases and thus the study was exempt from approval by ethics committees.

### Study population

We performed a retrospective analysis of de-identified data from pulmonary TB cases reported to the Brazilian Notifiable Diseases Information System (SINAN).

SINAN is a centralized system for the notification of transmissible diseases, including TB. Data stored in SINAN are maintained by the Brazilian Ministry of Health specifically by the DATASUS (the Information Technology Department of the Brazilian Unified Health System) and can be accessed through a file transfer protocol [6].

**Fig. 1:**
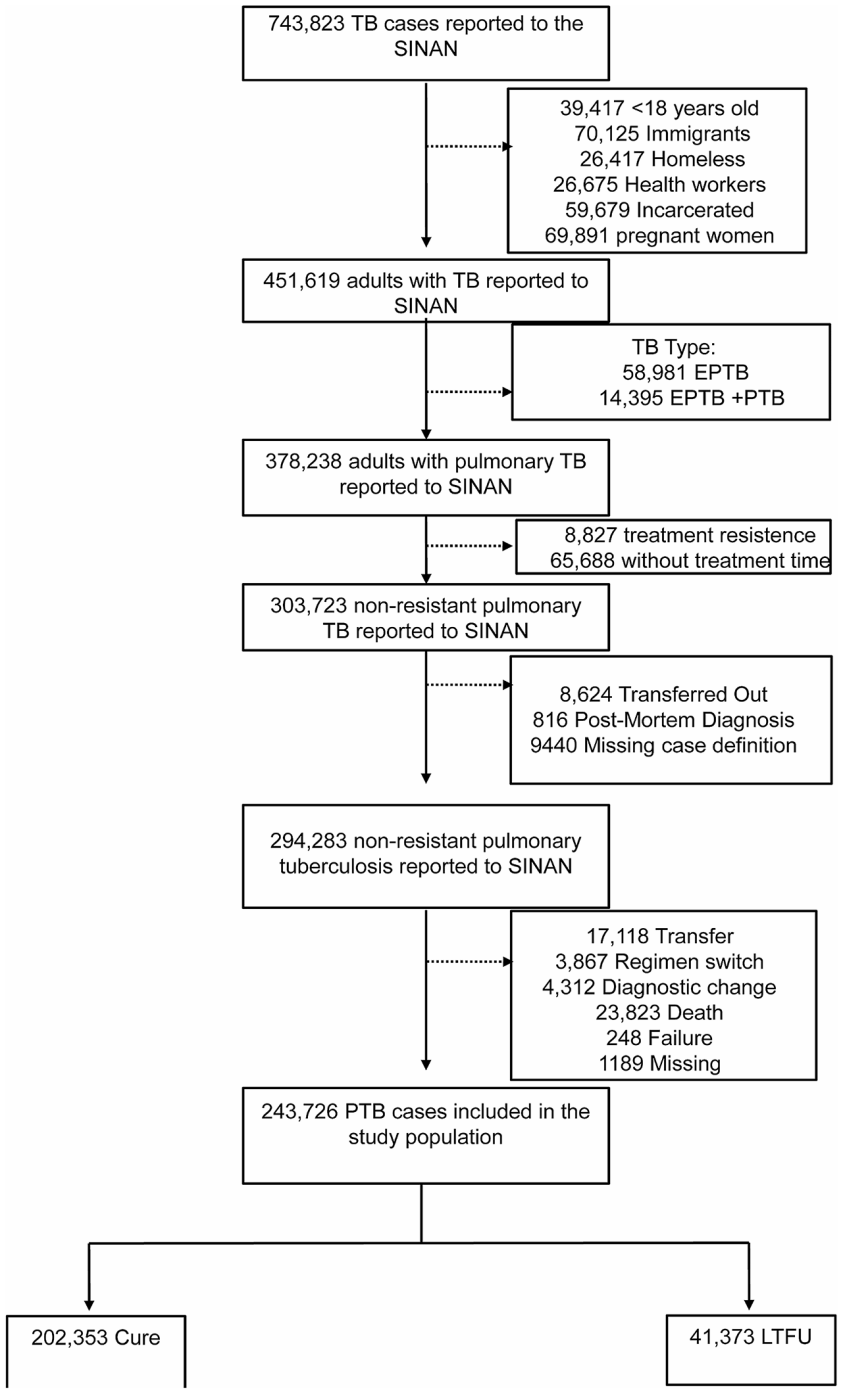
Flowchart demonstrating the study population. Abbreviations: TB: Tuberculosis; LTFU: Loss to follow up; EPTB: extrapulmonary tuberculosis; PTB: pulmonary tuberculosis; HCU: health care unit; .

We included in our study all individuals 18 years old or older, notified in SINAN with pulmonary TB from 2015 to 2022. We exclude from our study any patient that: (i) postmortem TB diagnosed; (ii) belongs to any special population (i.e. homelessness, liberty deprivation, pregnant, immigrants, and health worker), (iii) is resistant to any drug (rifampin, isoniazid, pyrazinamide, or ethambutol), (iv) outcome other than cure or LTFU, and with PTB and also had > = 1 EPTB site. **(**Fig. 1**).** Vulnerable populations were removed because they present a different pattern of risk of illness and LTFU than the general population.

### Variables definitions

The age variable was categorized using the following bins: children/teenage (0, 18], Young adult (18, 35], Adult (35, 50], Senior adult (50, 65] and Eldery > 80 years old. Biological sex: female or male, HIV infection: presence of an HIV diagnosis (self-reported); alcohol consumption: ever use of alcohol; tobacco use: ever smoking tobacco; drug use: ever use of drugs (including marijuana, cocaine, heroin or crack); race: self-reported races/ ethnicities, subdivided into Non-White (including “Yellow”, “Black”, “Pardo”, which defines mixed-race ancestry in Latin America [European, Indigenous and African], and Indigenous) and White; DOT: implementation of directly observed therapy; schooling: self-reported years of schooling. abnormal chest X-ray: thorax radiographic result indicative of TB; sensibility TB test: susceptible to all first-line drugs, resistant to any drug; smear grade: positive, negative, not performed. Comorbidities such as diabetes and mental illness were classified according to the presence or abstence in the moment of the TB diagnois (self-reported). Prior TB: patient report a history of TB treatment. This stratification was performed following criterion adopted by Brazilian Ministry of Health to report TB data [8].

### Data analyses

We divided our data analysis process into seven portions/steps: (i) descriptive analyses, (ii) data under sample, (iii) split data, (iv) feature elimination, (v) hyper-parameters tuning, (vi) model evaluation, and (vii) model building. To conduct descriptive analysis we used median followed by interval interquartile (IQR) to describe continuous variable and absolute and relative frequency to categorical. As our data could be considered imbalanced (i.e. ~3 cures for 1 LTFU) we performed an under sample of the most frequent class [9]. Hence, the data set resulting from this process has the same proportion of outcome (i.e. 1 cure for 1 LTFU), and then we split in train test data [10]. The training set was composed by 70% of the total data whereas 30% was kept for model evaluation. To reduce data dimensionality, we used Recursive Feature Elimination using Cross-Validation (RFECV) [11]. In this case, we selected RF as the estimator and used it in a 10-fold stratified cross-validation, then we selected the minimum number of variables that leads to the higher model accuracy following the elbow rule. To find the best set of parameters we used the grid search approach, thus for each model (i.e. Logistic Regression, Random Forest, and Light Gradient Boosting [12, 13]) we created a grid of parameters, in the train set we evaluated the best combination of the parameters. To select the best algorithm evaluation, we applied each model with its best combination of parameters to the test set. We then evaluate AUC, accuracy, sensitivity, and specificity [14, 15]. To understand the feature importance and feature contribution to each outcome on a global and local level we used Shapley values. The last step consisted of retraining the model using the whole data set [16, 17]. All codes are provide and could be checked at (https://github.com/rodriguesmsb/TBPrediction)

## Results

### Characteristics of the overall study population

Between, 2015, and 2022, 743,823 TB cases were notified in SINAN. 243,726 were included in the final study population, with 500,097 (~ 67%) of notifications removed according to our exclusion criteria (Fig. 1). The selected population was stratified according to 202,353 cases that experienced cure and 41,373 experienced LTFU (Fig. 1). At the time of the TB diagnosis, the LTFU group was younger (median age _LTFU_: 37.1 vs. _Cure_ 42.1 years), had more self-identified as non-white (_LTFU_ 72.8% vs. _Cure_65.3%), with lower schooling rates (≥ 12 years, _LTFU_ 2.33% vs. _Cure_6.38%) highest prevalence of HIV infection (_LTFU_ 13.2% vs. _Cure_5.99%) and prior TB (_LTFU_32.4% vs. _Cure_ 10.8%). Among consumption habits, the LTFU group presented the highest prevalence of all the consumption habits evaluated, such as alcohol use (_LTFU_ 29.0% vs. _Cure_16.1%), tobacco use (_LTFU_ 35.0% vs. _Cure_21.9%) and drug use (_LTFU_ 28.6% vs. _Cure_9.12%). Interestingly diabetes was less prevalent in LTFU group (_LTFU_ 5.67% vs. _Cure_10.9%). Noteworthy, the DOT was more prevalent among the cure group (_LTFU_ 21.2% vs. _Cure_ 41.4%). All the evaluated characteristics were statistically significant between the groups **(**Table 1**)**.

**Table 1:** Characteristics of the overall population of the study

Characteristics	Overall Population	Cure	Loss to follow-up	*p*-value
	*N* = 243,726	*N* = 202,353	*N* = 41,373	
**Age**				0.000
Children/ teenagers	4368 (1.79%)	3586 (1.77%)	782 (1.89%)	
Young Adult	95,083 (39.01%)	74,164 (36.65%)	20,919 (50.56%)	
Adult	69,117 (28.35%)	56,545 (27.94%)	12,572 (30.38%)	
Senior adult	52,216 (21.42%)	46,900 (23.17%)	5316 (12.84%)	
Elderly	3400 (1.39%)	3128 (1.54%)	272 (0.65%)	
Missing	19,542 (8.01%)	18,030 (8.91%)	1512 (3.65%)	
**Biologic Sex**:				< 0.001
Female	84,381(34.6%)	73,380(36.3%)	11,001(26.6%)	
Male	159,334(65.4%)	128,965(63.7%)	30,369(73.4%)	
Missing	11(0.00%)	8(0.00%)	3(0.01%)	
**Race**:				< 0.001
White	69,388(28.5%)	60,333(29.8%)	9055(21.9%)	
Non-white	162,314(66.6%)	132,193(65.3%)	30,121(72.8%)	
Missing	12,024(4.93%)	9827(4.86%)	2197(5.31%)	
**Schooling**				0.000
< 5 years	30,685(12.6%)	25,757(12.7%)	4928(11.9%)	
[5,9) years	57,397(23.5%)	45,487(22.5%)	11,910(28.8%)	
[9,12) years	74,104(30.4%)	62,998(31.1%)	11,106(26.8%)	
≥ 12 years	13,873(5.69%)	12,908(6.38%)	965(2.33%)	
Missing	67,667(27.8%)	55,203(27.3%)	12,464(30.1%)	
**Alcohol Use**	44,556(18.3%)	32,546(16.1%)	12,010(29.0%)	0.000
Missing	8439(3.46%)	6656(3.29%)	1783(4.31%)	
**Diabetes**:	24,398(10.0%)	22,054(10.9%)	2344(5.67%)	< 0.001
Missing	8850(3.63%)	6875(3.40%)	1975(4.77%)	
**Mental Illness**	5961(2.45%)	4797(2.37%)	1164(2.81%)	< 0.001
Missing	9259(3.80%)	7242(3.58%)	2017(4.88%)	
**Drug Use**	30,290(12.4%)	18,445(9.12%)	11,845(28.6%)	0.000
Missing	9846(4.04%)	7765(3.84%)	2081(5.03%)	
**HIV infection**	17,575(7.21%)	12,124(5.99%)	5451(13.2%)	0.000
Missing	35,611(14.6%)	27,091(13.4%)	8520(20.6%)	
**Prior TB**	35,279(14.5%)	21,858(10.8%)	13,421(32.4%)	0.000
**Tobacco Use**:	58,705(24.1%)	44,233(21.9%)	14,472(35.0%)	0.000
Missing	8704(3.57%)	6842(3.38%)	1862(4.50%)	
**DOT**	92,539(38.0%)	83,778(41.4%)	8761(21.2%)	0.000
Missing	48,856(20.0%)	37,263(18.4%)	11,593(28.0%)	

***Table note***: Data represent no. (%), except for age, which is presented as median and interquartile range (IQR).

Definition of age: children/teenage (0, 18], young adult (18, 35], Adult (35, 50], Senior adult (50, 65] and Eldery > 80 years old

Definition of alcohol use: Past or current any consumption of alcohol

Definition of smoking: Past or current smoking of tobacco.

Definition of non-white race: combination of black, mixed, pardo, yellow and indigenous.

Definition of drug use: Past or current drug use (marijuana, cocaine, heroin, or crack).

Other comorbidities: Include cancer, kidney disease, chronic obstructive pulmonary disease, emphysema, allergies, and asthma.

Abbreviations: TB: tuberculosis; PTB: Pulmonary tuberculosis; DOT: Directly Observed Therapy; EPTB: Extrapulmonary tuberculosis.

### Comparing machine learning algorithms to predict LTFU

**Fig. 2:**
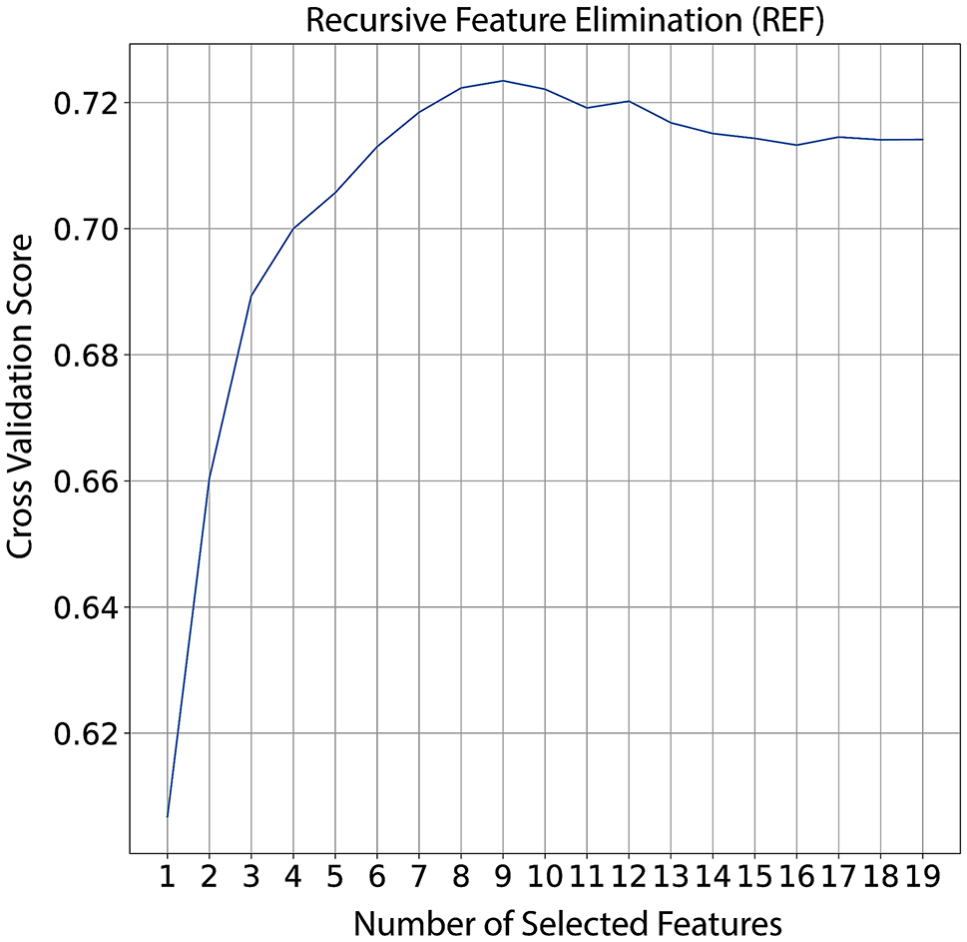
Recursive feature selection elimination. In the x-axis indicating the number of features used by the model while in the y-axis indicating the AUC achieved during the cross-validation

We initiated our model development with 13 variables of which 8 were selected as the most informative by our RFECV approach **(**Fig. 2**)**: (i) schooling, (ii) sex, (iii) prior TB, (iv) HIV infection, (v) alcohol use, (vi) drug use, (vii) tobacco use and (vii) age. To predict those patients who are more likely to experience an LTFU we proposed three different models using the variables listed above. In our investigation into predicting patient outcomes, three diverse models were employed, each revealing unique hyperparameter preferences for optimal performance. The logistic regression model demonstrated its peak predictive capabilities with a strong regularization, notably C = 0.01. This underscored the critical role of regularization strength in striking a balance between model complexity and generalization. The RF model achieved its best performance by setting the maximum depth to 8, which means each of the model’s decision trees is allowed to make decisions down to eight levels deep. Additionally, it used an ensemble of 500 decision trees, meaning the model’s final prediction is based on the combined output of 500 trees. This setup highlights the critical importance of these specific settings—both the depth of decision-making in each tree and the total number of trees in the ensemble—for improving the model’s ability to accurately predict outcomes. In the case of the Light Gradient Boosting model, optimal performance was achieved with trees of max depth 4, 500 decision trees (no. of estimators), and a learning rate of 0.01. These results highlighted the intricate interplay between tree complexity, ensemble size, and the learning rate in achieving superior predictive capabilities.

The next phase consisted of evaluating the three models (using the parameters described above) on the test set. In this case, we found that classifiers presented similar results (Supplementary Table S1).

**Fig. 3:**
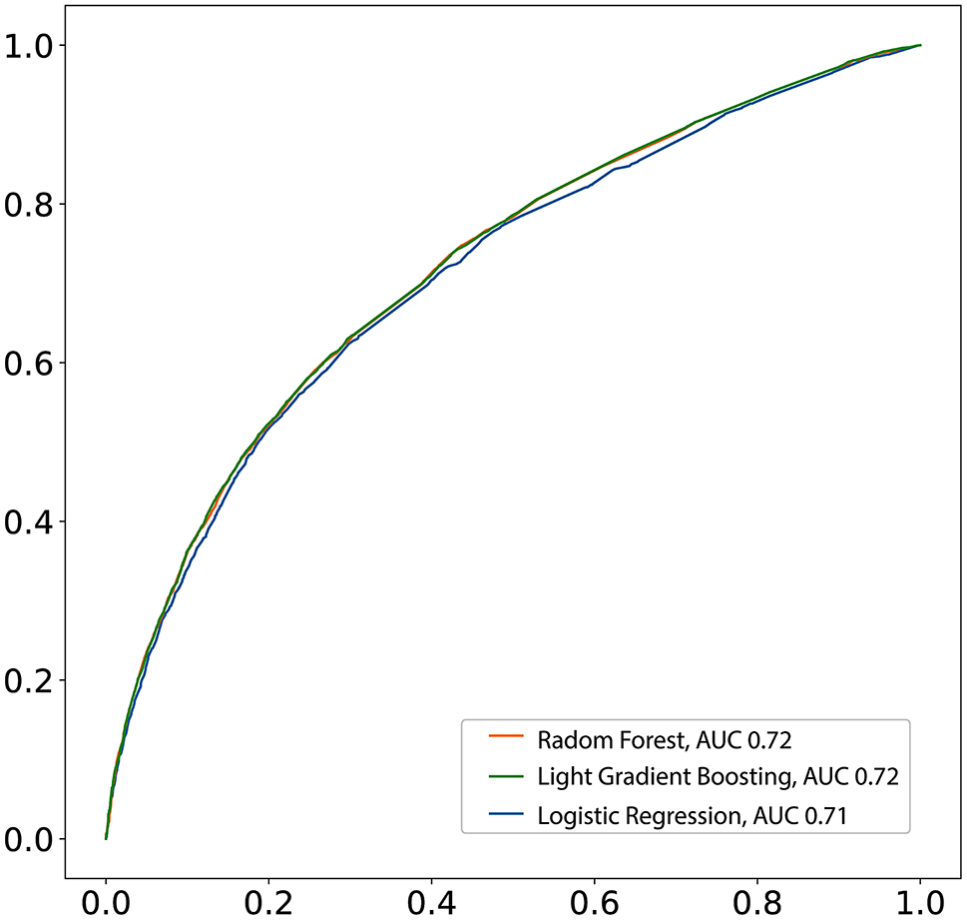
Receiver operating characteristic curve (ROC) for prediction of LTFU based on data available in SINAN using three different Machine Learning algorithm

**Fig. 4:**
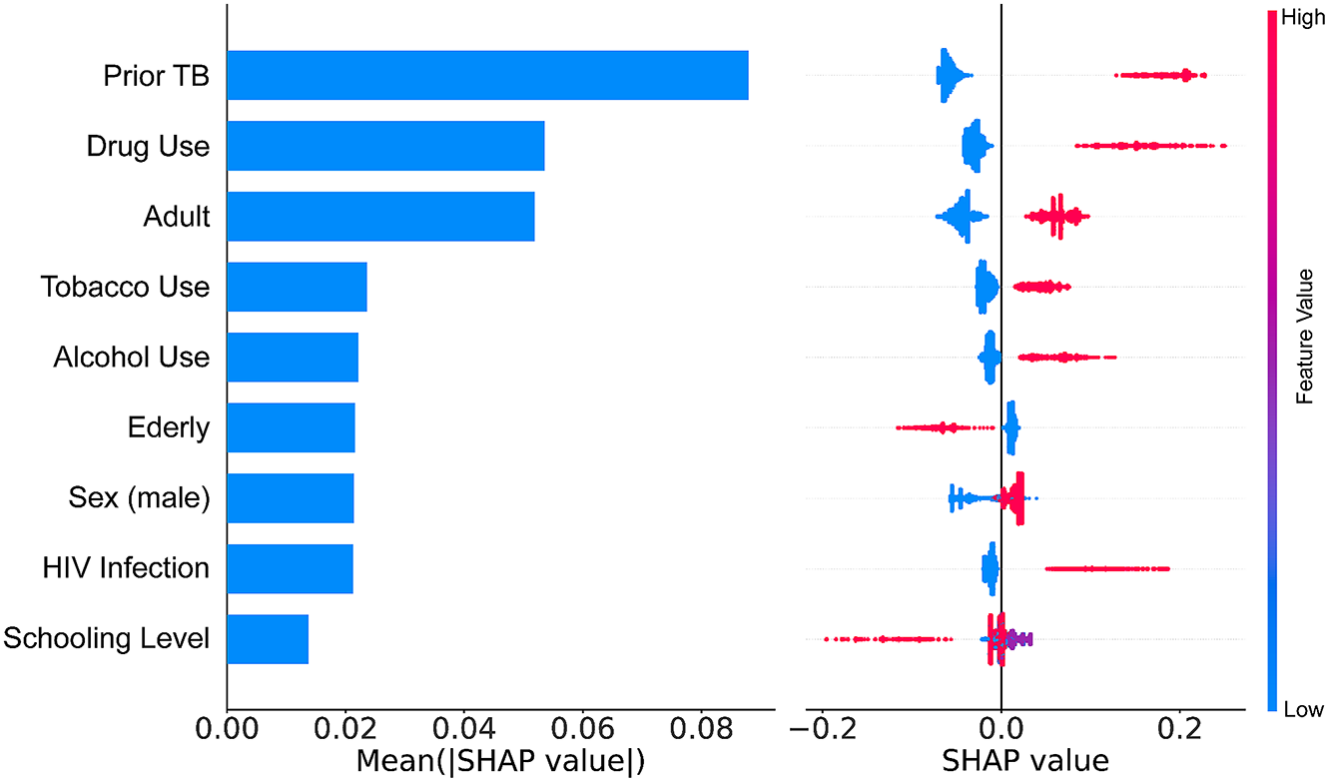
Feature importance computed using SHAP-values on test set. and Relationship between feature value and treatment outcome. Blue dot indicates Cure and, for categorical features the value of no. Red dots indicating LTFU patients and, for categorical features the value of Yes

According to our calibration plot, the Light Gradient Boosting presented the best result since the predicted probability of an LTFU corresponds to the true likelihood of the positive class being true (Supplementary Fig. S1). The Random Forest presented the worst result. In this case, the model probability underestimated the real likelihood of the positive class. Thus based, on all the results we found, we decided to use the Light Gradient Boosting to construct our predictive model (Fig. 3). We used SHAP values to allocate the contribution of each feature to a model’s prediction, offering insights into feature importance and interactions. Such values help interpret complex models, providing a nuanced understanding of the factors influencing specific predictions. According to our model, previous TB was the most important feature. In this case, a patient who experienced prior TB had increased likelihood to evolve to LTFU. Another important feature was drug use. Patients who reported to use drugs had the probability of evolve to LTFU during an ATT increased **(**Fig. 4**)**.

## Discussion

In this study of pulmonary TB cases reported to SINAN in Brazil, we developed a risk score that effectively stratified before treatment initiation those TB cases at higher risk of LTFU during ATT. Our score used data from 7 features, all of which were from the case notification form, and were publicly available. Those features included clinical and epidemiologic information, that can be collected by health professionals before treatment initiation, and which predicted LTFU independent of other characteristics. The use of this risk score could potentially provide crucial information to target specific patients since the diagnosis and improve the successful ATT completion, potentially facilitating the achievement of the WHO target of 90% of patients with treatment success [18].

Importantly, in our study, 14.5% of the total population experienced LTFU, which represents an important problem for public health because of the risk of *M. tuberculosis* transmission; drug-resistant strains can also be generated [19]. Importantly, the rates of DOT in the group that experienced the LTFU were significantly lower than the cure group. Enhancing the importance of the detection of these patients at the beginning of TB treatment might help clinicians in choosing priorities for DOT and the target populations for the Brazilian national TB program.

Our probabilistic score was developed using clinical and sociodemographic data readily collected in most clinical care settings, even in resource-limited settings. Among the variables selected, prior TB, consumption habits (alcohol, tobacco, or drug use), age (adult and elderly), biological sex, HIV infection, and schooling level were the risk factors that most contributed to an LTFU during TB treatment. Some of these characteristics have been explored and linked to unfavorable TB treatment outcomes through the relationship with poor therapy adherence, LTFU, and treatment discontinuation [20–27]. It is important to highlight that our study identified history of prior TB as the variable with the most significant impact on the model’s ability to predict LTFU. This finding is consistent with extensive literature, which attributes this impact to a mix of psychological factors, barriers to healthcare access, social conditions, and stigma [28–31]. . Additionally, a study using the SINAN database highlighted that a history of previous treatment abandonment is the primary risk factor for LTFU in new treatment cycles, underlining the importance of past treatment adherence in predicting and managing future outcomes [32]. .

In a previous study, a similar score was developed to predict unfavorable anti-TB treatment outcomes in people living with diabetes from China, however using clinical and radiologic data [23]. Another study from Mexico developed an algorithm to predict mortality, failure, and drug resistance in newly diagnosed TB patients with clinical features and laboratory tests [27]. In contrast, our score could be applied in patients with or without diabetes, by utilizing only clinical information, without the necessity of laboratory data or radiographic exams.

While exploring data from the RePORT-Brazil consortium, we have previously reported a clinical prediction model for unfavorable pulmonary TB treatment outcomes [20]. That score utilized information that was not readily available in SINAN, thus we found it difficult to translate to the nationwide TB program in Brazil. The present study intended to create a score that could be employed in all settings, especially in those with limited resources, which could certainly help guide interventions at the moment of diagnosis, before starting treatment in a large country such as Brazil.

Our risk model had several limitations. First, the study utilized nationwide public data, and several features had missing data and were exposed to a wide range of demographic and regional discrepancies. Second, most co-morbidities and clinical characteristics were self-reported, which may provide potential misclassification bias. The study included only pulmonary TB cases and consequently may not be applied to extrapulmonary or disseminated TB. Also, we excluded vulnerable populations, and the total number of exclusions were higher than 50% of the total cases reported limiting the use in similar populations to those included in our study. We suggest that future scores include more clinical data, physical exam, and social economic conditions to improve the accuracy and extend the applicability in clinical practice.

Despite the limitations, to the best of our knowledge, this is the first prognostic score model developed in South America using only clinical and epidemiologic data from disease notification forms, obtained before therapy initiation, with relatively accurate prediction. The resulting model is parsimonious and should be utilized by clinicians through a nomogram or web application (https://tbprediction.onrender.com), assisting in TB care and potentially improving the successful completion of ATT of pulmonary TB patients.

### Electronic supplementary material

Below is the link to the electronic supplementary material.

Supplementary Material 1

## Data Availability

All data accessed in this study were obtained from a publicly available platform and pre-processed by the Brazilian Ministry of Health (https://datasus.saude.gov.br). All generated and/or analyzed during the current study are available in the github repository, available in the link: https://github.com/rodriguesmsb/TBPrediction.

## Declarations

### Disclaimer

The funders had no role in study design, data collection and analysis, decision to publish, or preparation of the manuscript.
