# Supplementary material for: Machine learning algorithms using national registry data to predict loss to follow-up during tuberculosis treatment
Source: BMC Public Health. 2024 May 23;24:1385. doi: 10.1186/s12889-024-18815-0 (PMC11112756; doi:10.1186/s12889-024-18815-0)

Machine learning algorithms using national registry data to predict loss to follow-up during tuberculosis treatment

Moreno M. S. Rodrigues; Beatriz Barreto-Duarte; Caian L. Vinhaes; Mariana Araújo-Pereira; Eduardo R. Fukutani; Keityane Bone Bergamaschi; Afrânio Kristki; Marcelo Cordeiro-Santos; Valeria C. Rolla; Timothy R. Sterling; Artur T. L. Queiroz; Bruno B. Andrade; for the RePORT Brazil consortium

Supplementary Material

Content

1. Supplementary Table 1
2. Supplementary Figure 1

**Supplementary table 1 Comparison between the models**. Abbreviations: PPV: Positive predictive value; NPV: Negative predictive value

| **Model** | **Accuracy** | **Sensitivity** | **Specificity** | **PPV** | **NPV** |
| --- | --- | --- | --- | --- | --- |
| Logistic Regression | 0.67 | 0.58 | 0.75 | 0.70 | 0.64 |
| Random Forest | 0.67 | 0.62 | 0.60 | 0.69 | 0.66 |
| Light Gradient Boosting | 0.67 | 0.62 | 0.72 | 0.69 | 0.66 |

**Supplementary Figure 1** **Calibration plot.** The x-axis indicating the probability of been predicted as LTFU by the model while the y-axis shows the observed probability. Black line indicates a perfect relation about predicted and observed probability. Blue lines indicate the relation between predicted and observed probability or (a) LightGradient Boosting, (b) Random Forest and (c) Logistic regression


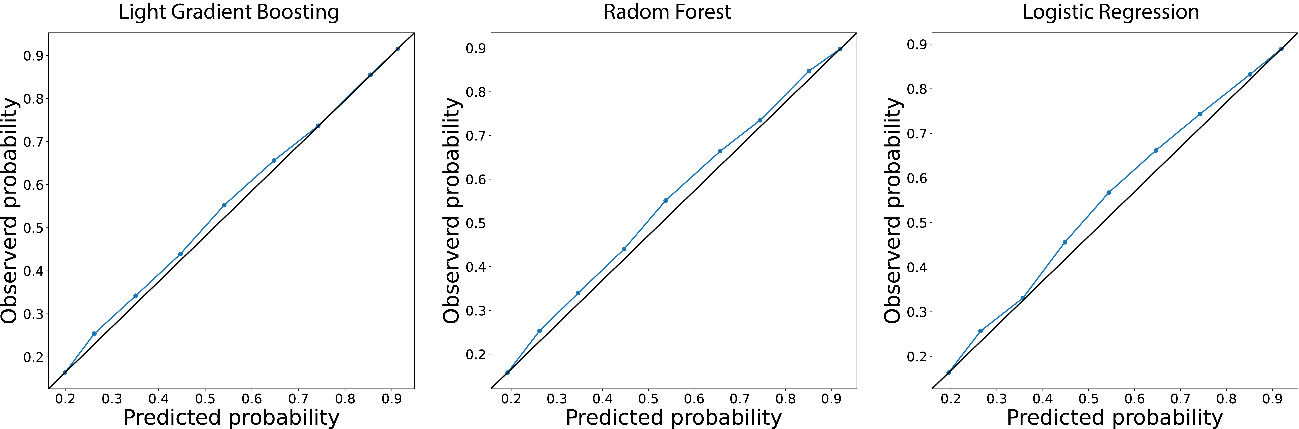

Supplement: Supplementary file 1 — Supplementary Material 1 [file 12889_2024_18815_MOESM1_ESM.docx]
